# Phenolic-Enriched Ethyl Acetate Fraction of Chatuphalatika Inhibits HMG-CoA Reductase and Preferentially Improves Hepatic Metabolic Parameters in High-Fat Diet-Fed Mice

**DOI:** 10.3390/molecules31122184

**Published:** 2026-06-22

**Authors:** Salin Mingmalairak, Prasob-orn Rinthong

**Affiliations:** 1 Department of Physiology, Faculty of Medicine, Chiang Mai University, Chiang Mai 50200, Thailand; salin.mingmalairak@cmu.ac.th; 2 Pharmaceutical Chemistry and Natural Product Research Unit, Faculty of Pharmacy, Mahasarakham University, Maha Sarakham 44150, Thailand

**Keywords:** Chatuphalatika, polyphenols, metabolic syndrome, HMG-CoA reductase, dyslipidemia, hepatic steatosis, natural products

## Abstract

Chatuphalatika is a traditional Thai polyherbal formulation whose metabolically active fraction has not been identified. This study fractionated the aqueous extract (CPT) by sequential liquid–liquid partitioning to obtain solvent fractions. The ethyl acetate fraction (CPTX) had the highest total phenolic content and was enriched in hydrolyzable tannins, particularly chebulagic acid. CPTX showed the strongest inhibitory activity against HMG-CoA reductase in vitro. In vivo, C57BL/6 mice were fed a high-fat diet for 12 weeks, then treated with CPT, CPTX, or silymarin for 8 weeks while high-fat diet feeding continued. Both CPT and CPTX improved serum lipid profiles. High-dose CPTX (500 mg/kg) additionally reduced fasting blood glucose, serum ALT, and relative liver weight, without affecting body weight or adipose tissue weights. These findings indicate that phenolic enrichment concentrates the hepatic and lipid-lowering activity of Chatuphalatika. HMG-CoA reductase inhibition was used as a screening criterion to identify CPTX as the active fraction; the in vivo hepatometabolic improvements are consistent with, but do not directly confirm, modulation of cholesterol biosynthesis and hepatic lipid metabolism.

## 1. Introduction

Metabolic syndrome (MetS) is a cluster of metabolic disturbances including dyslipidemia, impaired glucose homeostasis, hypertension, and central obesity, collectively associated with increased risk of cardiovascular disease, type 2 diabetes, and nonalcoholic fatty liver disease (NAFLD) [1,2]. The liver has a central role in lipid and glucose regulation, and excessive dietary fat intake promotes hepatic lipid accumulation and metabolic dysfunction, making it a key target in MetS management [2,3].

HMG-CoA reductase is the rate-limiting enzyme in cholesterol biosynthesis and the primary target of statins [4,5]. While statins effectively reduce cardiovascular risk [6], MetS often involves multiple metabolic disturbances beyond dyslipidemia, including impaired glucose metabolism and hepatic dysfunction [1,2]. This has driven interest in polyphenol-rich herbal preparations as complementary agents with potentially broader metabolic effects [7,8].

Plant polyphenols, including hydrolyzable tannins such as gallic acid, ellagic acid, chebulagic acid, and chebulinic acid, have been reported to modulate hepatic lipid accumulation, cholesterol metabolism, glucose homeostasis, and inflammation [7,8,9]. In tannin-rich polyherbal formulations, the combined phytochemical profile may contribute to metabolic effects that extend beyond any single constituent.

Chatuphalatika is a traditional Thai polyherbal formulation used in Thai traditional medicine. It consists of equal proportions of the fruits of *Terminalia chebula* Retz. (Combretaceae), *Terminalia bellirica* (Gaertn.) Roxb. (Combretaceae), *Terminalia arjuna* Wight & Arn., and *Phyllanthus emblica* L. (Phyllanthaceae). This formulation is conceptually related to Triphala, a well-known Ayurvedic formulation composed of *T. chebula*, *T. bellirica*, and *P. emblica*, but represents a distinct Thai traditional formula [10,11,12]. Triphala and its constituent fruits have been shown to modulate lipid and glucose metabolism [10,13,14], and Chatuphalatika has demonstrated antioxidant, anti-inflammatory, antihyperuricemic, and lipid-lowering activity [11,15]. The constituent fruits are rich in hydrolyzable tannins and phenolic acids, consistent with their reported phytochemical activities [9,11,12,16,17].

Our previous work showed that Chatuphalatika aqueous extract reduced lipid levels and adiposity in HFD-fed mice [15]. However, the metabolically active fraction of the crude decoction has not been identified. Fractionation guided by bioactivity is a practical approach to locate the active constituents and determine whether enriching a specific phytochemical group improves biological activity.

This study investigated the metabolic effects of Chatuphalatika aqueous extract (CPT) and its solvent fractions, with the aim of determining whether phenolic enrichment enhances metabolic activity. HMG-CoA reductase inhibition was used as a screening criterion to identify the most active fraction, which was then evaluated alongside CPT in a high-fat diet-induced MetS mouse model, focusing on dyslipidemia, fasting glucose, and hepatic metabolic parameters.

## 2. Results

### 2.1. Phytochemical Composition of CPT and Its Solvent Fractions

Chatuphalatika aqueous extract (CPT) was prepared by decoction, corresponding to the traditional method of preparation. The obtained CPT appeared as a dark brown powder, with an extraction yield of 22.86% and a total phenolic content of 363.0 ± 13.33 mg GAE/g extract. HPLC analysis identified gallic acid and chebulagic acid as the predominant phenolic constituents, with concentrations of 99.8 ± 5.62 mg/g and 50.9 ± 0.94 mg/g extract, respectively (Table 1).

**Figure 1:**
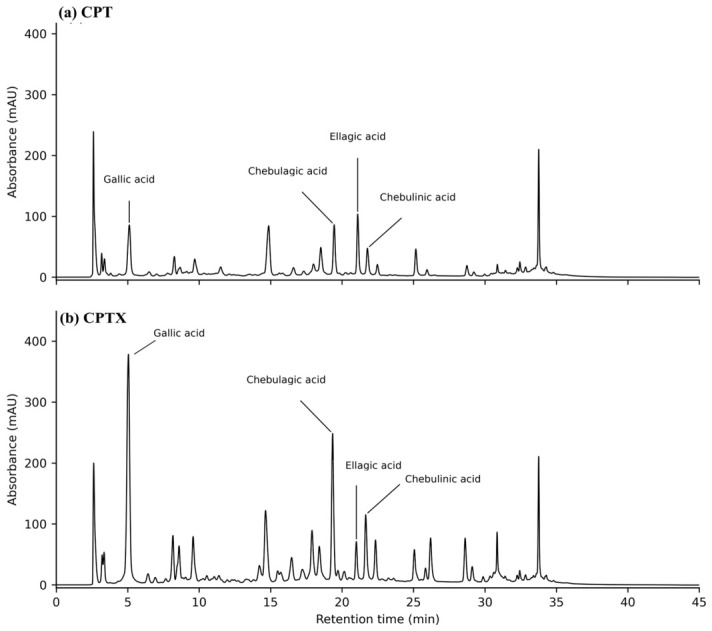
HPLC chromatograms of Chatuphalatika aqueous extract (CPT) and its ethyl acetate fraction (CPTX) detected at 270 nm. (**a**) CPT; (**b**) CPTX. Major phenolic constituents are indicated: gallic acid, chebulagic acid, ellagic acid, and chebulinic acid.

Sequential solvent partitioning of CPT produced fractions with distinct phytochemical profiles. The ethyl acetate fraction, designated as CPTX, had the highest total phenolic content at 458.8 ± 25.96 mg GAE/g extract and was particularly enriched in chebulagic acid at 105.5 ± 4.72 mg/g extract, approximately two-fold higher than in CPT. The chloroform fraction contained low phenolic content, the aqueous fraction showed moderate content, and the hexane fraction contained no detectable phenolic compounds. These results indicate that ethyl acetate partitioning selectively concentrated the phenolic constituents of Chatuphalatika, particularly hydrolyzable tannins. The representative HPLC chromatograms of CPT and CPTX (Figure 1) illustrate the distinct phenolic profiles of the two preparations, with CPTX showing a markedly more complex pattern and more prominent chebulagic acid peak.

### 2.2. HMG-CoA Reductase Inhibitory Activity

**Figure 2:**
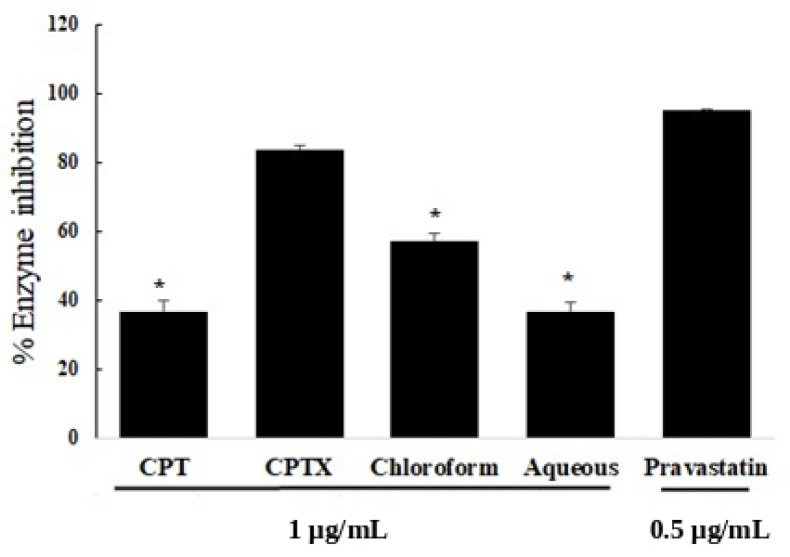
HMG-CoA reductase inhibitory activity of CPT and its solvent fractions. Inhibitory activity of Chatuphalatika aqueous extract (CPT), its solvent fractions, and pravastatin against HMG-CoA reductase. CPT and its fractions were tested at 1 µg/mL, whereas pravastatin was tested at 0.5 µg/mL. Data are expressed as mean ± SD (n = 3). Statistical significance was determined using one-way ANOVA followed by Tukey’s post hoc test. * *p* < 0.05, significantly lower than pravastatin.

CPT and its solvent fractions were evaluated for inhibitory activity against HMG-CoA reductase, the rate-limiting enzyme in cholesterol biosynthesis. Among the tested samples, CPTX at 1 µg/mL exhibited the strongest inhibitory activity at 83.9 ± 1.24%, while CPT and the other fractions showed lower inhibitory activity ranging from 36.6% to 57.1% at the same concentration (Figure 2). Pravastatin, included as a positive control at 0.5 µg/mL, showed 95.04 ± 0.45% inhibition. These results indicate that phenolic enrichment in CPTX was associated with enhanced HMG-CoA reductase inhibition, consistent with its enrichment in hydrolyzable tannins, particularly chebulagic acid.

### 2.3. Establishment of the HFD-Induced Metabolic Syndrome Mouse Model

**Figure 3:**
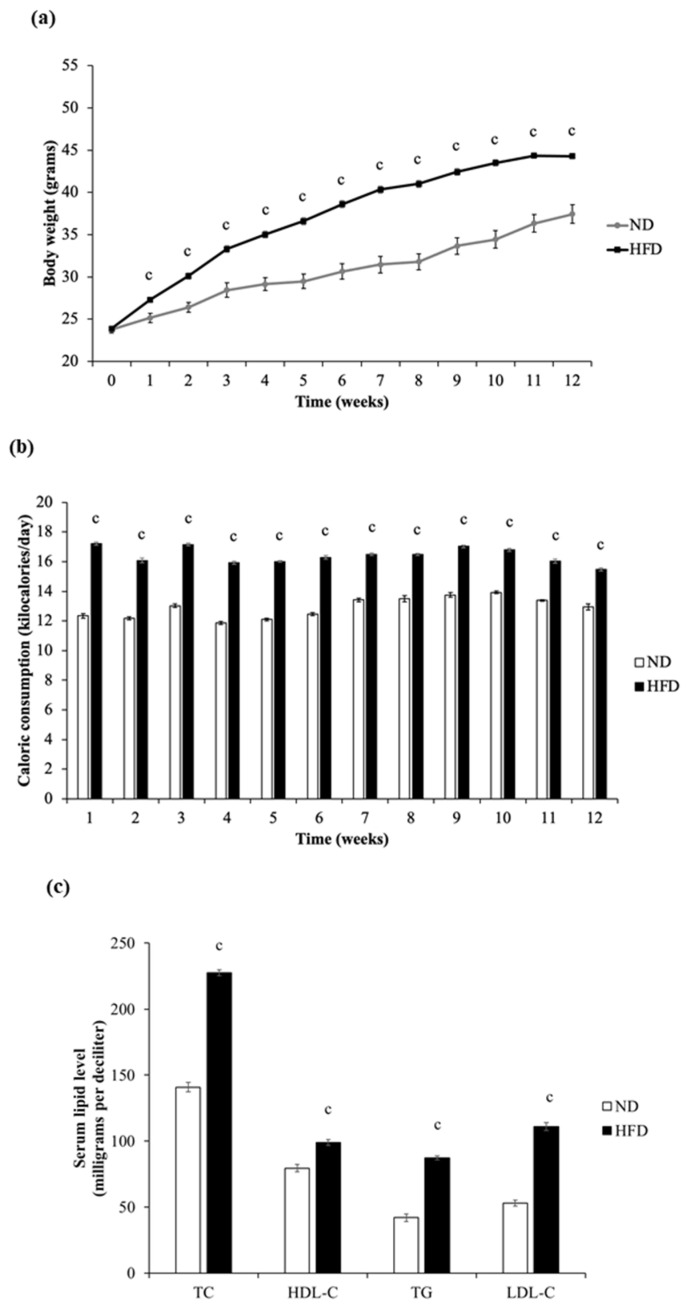
Body weight and caloric intake during HFD induction. (**a**) Body weight, (**b**) caloric consumption, and (**c**) plasma lipid levels of mice fed a normal diet (ND) or high-fat diet (HFD) for 12 weeks. Data are expressed as mean ± SEM (n = 10 for ND group and n = 50 for HFD group, representing all HFD-assigned mice prior to treatment group allocation). Statistical significance was analyzed using Student’s *t*-test. ^c^ indicates *p* < 0.001 versus the ND group.

Mice were fed either a normal diet (ND) or high-fat diet (HFD) for 12 weeks before treatment initiation. HFD feeding induced a significant increase in body weight from week 1 through week 12 (Figure 3a), with a final body weight 18.43% higher than ND-fed mice. Caloric consumption was consistently higher in the HFD group throughout the induction period (Figure 3b). An assessment of plasma lipid levels revealed significant increases across all lipid parameters in HFD-fed mice compared to ND-fed mice (Figure 3c). These results indicated that HFD feeding induced obesity-related changes before treatment initiation.

### 2.4. Effects of CPT and CPTX on Body Weight and Caloric Consumption in HFD-Fed Mice

**Figure 4:**
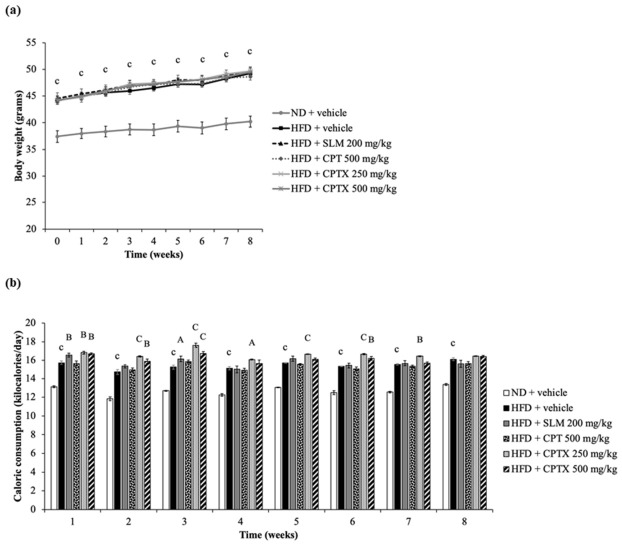
Effects of CPT and CPTX on body weight and caloric intake during the treatment period. (**a**) Body weight and (**b**) caloric consumption of HFD-fed mice treated with vehicle, silymarin (SLM, 200 mg/kg), CPT (500 mg/kg), or CPTX (250 or 500 mg/kg) for 8 weeks. Data are expressed as mean ± SEM (n = 10). Statistical significance was analyzed using Student’s *t*-test or one-way ANOVA followed by the Student-Newman-Keuls post hoc test, as appropriate. ^c^ *p* < 0.001 versus the ND + vehicle group. ^A^, ^B^, and ^C^ indicate *p* < 0.05, *p* < 0.01, and *p* < 0.001 versus the HFD + vehicle group, respectively.

After 12 weeks of diet induction, HFD-fed mice continued on the HFD and were orally treated with vehicle, SLM (200 mg/kg), CPT (500 mg/kg), or CPTX (250 or 500 mg/kg) for an additional 8 weeks. ND-fed mice were maintained on the ND and received vehicle treatment during the same period. Vehicle-treated HFD-fed mice maintained significantly higher body weight and caloric consumption than ND-fed mice during the treatment period (Figure 4a,b). Treatment with SLM, CPT, or CPTX did not significantly alter body weight in HFD-fed mice relative to vehicle-treated HFD mice (Figure 4a). Caloric consumption fluctuated during the treatment period with no consistent dose-dependent reduction among the treatment groups (Figure 4b). CPT and CPTX therefore did not reduce body weight under continued HFD feeding conditions.

### 2.5. Effects of CPT and CPTX on Relative Liver and Adipose Tissue Weights

**Figure 5:**
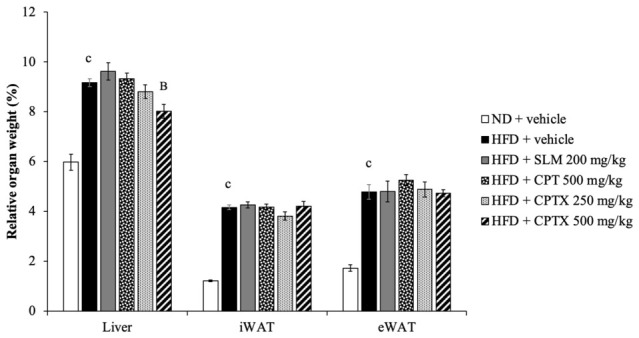
Effects of CPT and CPTX on relative organ weights in HFD-fed mice. Relative weight of liver, inguinal white adipose tissue (iWAT), and epididymal white adipose tissue (eWAT) in HFD-fed mice treated with vehicle, silymarin (200 mg/kg), CPT (500 mg/kg), or CPTX (250 or 500 mg/kg). Data are expressed as mean ± SEM (n = 10). Statistical significance was analyzed using one-way ANOVA followed by the Student-Newman-Keuls post hoc test. ^c^ *p* < 0.001 versus the ND + vehicle group; ^B^ *p* < 0.01 versus the HFD + vehicle group.

Vehicle-treated HFD-fed mice exhibited significantly higher relative weights of the liver, inguinal white adipose tissue (iWAT), and epididymal white adipose tissue (eWAT) than ND-fed mice (Figure 5). Notably, treatment with CPTX at 500 mg/kg significantly reduced relative liver weight in HFD-fed mice compared with vehicle-treated HFD mice. However, CPTX did not significantly alter the relative weights of iWAT or eWAT. These results suggest that high-dose CPTX preferentially improved liver-associated changes without markedly affecting adipose tissue mass.

### 2.6. Effects of CPT and CPTX on Fasting Blood Glucose

**Figure 6:**
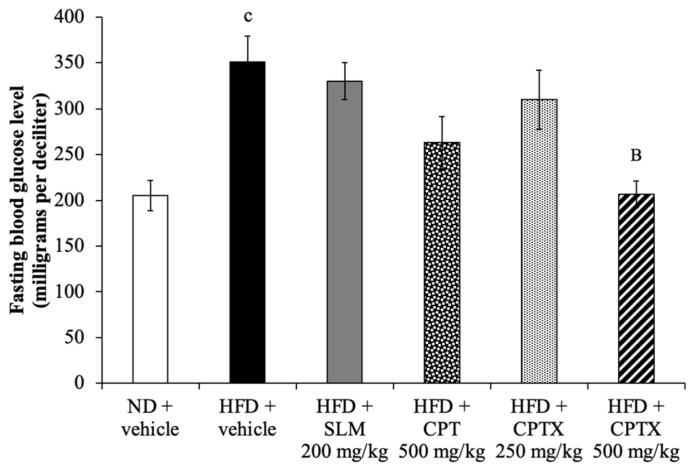
Effect of CPT and CPTX on fasting blood glucose in HFD-fed mice. Fasting blood glucose levels of HFD-fed mice treated with vehicle, silymarin (200 mg/kg), CPT (500 mg/kg), or CPTX (250 or 500 mg/kg). Data are expressed as mean ± SEM (n = 10/group). Statistical significance was analyzed using one-way ANOVA followed by the Student-Newman-Keuls post hoc test. ^c^ indicates *p* < 0.001 versus the ND + vehicle group, and ^B^ indicates *p* < 0.01 versus the HFD + vehicle group.

Fasting blood glucose levels were significantly elevated in vehicle-treated HFD mice compared with ND-fed mice, indicating the development of glucose metabolic disturbance in the HFD model (Figure 6). Treatment with CPTX at 500 mg/kg significantly reduced fasting blood glucose levels compared with vehicle-treated HFD mice. In contrast, the lower dose of CPTX and CPT showed no significant glucose-lowering effect under the present experimental conditions. These findings indicate that high-dose CPTX improved fasting glucose disturbance in HFD-fed mice.

### 2.7. Effects of CPT and CPTX on Serum Lipid Profiles

**Figure 7:**
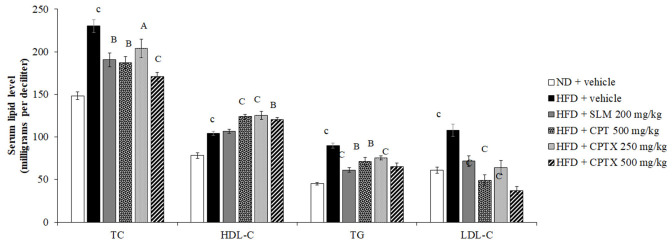
Effects of CPT and CPTX on serum lipid profiles in HFD-fed mice. Serum lipid levels of HFD-fed mice treated with vehicle, silymarin (200 mg/kg), CPT (500 mg/kg), or CPTX (250 or 500 mg/kg). Data are expressed as mean ± SEM (n = 10/group). Statistical significance was analyzed using one-way ANOVA followed by the Student-Newman-Keuls post hoc test. ^c^ *p* < 0.001 versus the ND + vehicle group; ^A^, ^B^, and ^C^ indicate *p* < 0.05, *p* < 0.01, and *p* < 0.001 versus the HFD + vehicle group, respectively.

Vehicle-treated HFD-fed mice exhibited significant dyslipidemia, as demonstrated by increased serum levels of total cholesterol (TC), triglycerides (TG), low-density lipoprotein cholesterol (LDL-C), and high-density lipoprotein cholesterol (HDL-C) compared with ND-fed mice (Figure 7). Treatment with SLM (200 mg/kg) significantly reduced TC, TG, and LDL-C levels in HFD-fed mice compared with vehicle-treated HFD mice. Similarly, CPT at 500 mg/kg and CPTX at both 250 and 500 mg/kg significantly decreased TC, TG, and LDL-C levels. In addition, CPT and CPTX significantly increased HDL-C levels compared with vehicle-treated HFD mice. These findings demonstrate that both CPT and CPTX improved HFD-induced dyslipidemia, with CPTX showing consistent lipid-modulating effects at both tested doses.

### 2.8. Effects of CPT and CPTX on Serum Transaminase Levels

**Figure 8:**
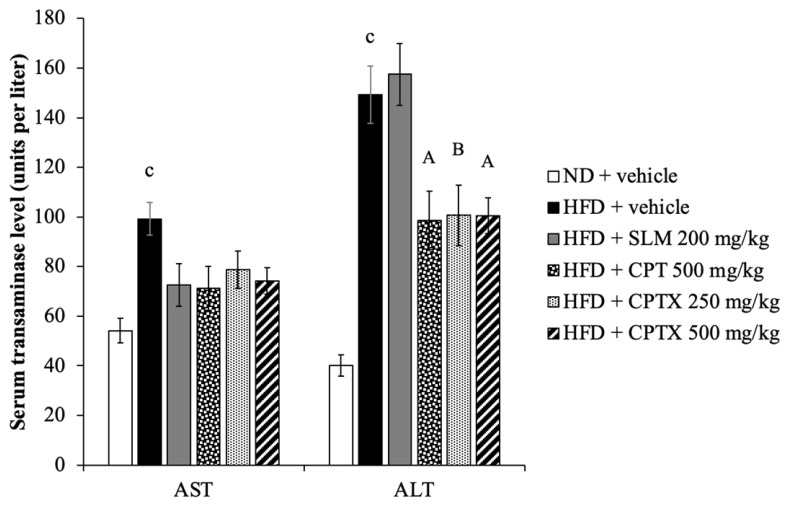
Effects of CPT and CPTX on serum transaminase levels in HFD-fed mice. Serum transaminase levels (ALT and AST) of HFD-fed mice treated with vehicle, silymarin (200 mg/kg), CPT (500 mg/kg), or CPTX (250 or 500 mg/kg). Data are expressed as mean ± SEM (n = 10/group). Statistical significance was analyzed using one-way ANOVA followed by the Student-Newman-Keuls post hoc test. ^c^ *p* < 0.001 versus the ND + vehicle group; ^A^ and ^B^ indicate *p* < 0.05 and *p* < 0.01 versus the HFD + vehicle group, respectively.

Serum alanine transaminase (ALT) and aspartate transaminase (AST) levels were markedly elevated in vehicle-treated HFD-fed mice compared with ND-fed mice, indicating HFD-induced liver injury or hepatic metabolic stress (Figure 8). Treatment with CPT (500 mg/kg) and CPTX at both 250 and 500 mg/kg significantly reduced serum ALT levels compared with vehicle-treated HFD mice. The reduction in AST was less pronounced. CPT and CPTX therefore attenuated HFD-induced ALT elevation, consistent with a hepatoprotective effect.

### 2.9. Gross Morphology and Visceral Fat Distribution at Necropsy

**Figure 9:**
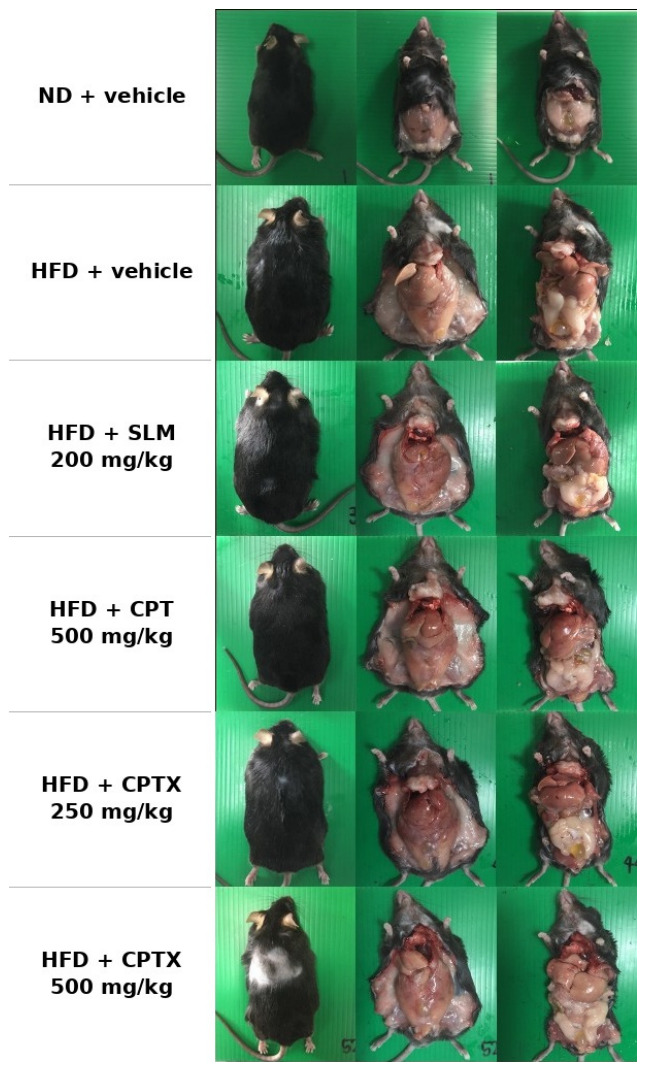
Representative gross morphology and visceral fat distribution at necropsy. Representative photographs of one animal per experimental group at the end of the 8-week treatment period. Each row shows the same animal in dorsal view (**left**), ventral view with intact abdominal cavity (**center**), and ventral view with exposed visceral compartment (**right**). HFD + vehicle mice showed markedly increased visceral fat in the inguinal and epididymal depots compared with ND + vehicle mice, consistent with persistence of the obese phenotype at necropsy. CPT, silymarin (200 mg/kg), and low-dose CPTX (250 mg/kg) did not visibly reduce adipose accumulation. High-dose CPTX (500 mg/kg) showed reduced visceral fat deposition, consistent with the significant reduction in relative liver weight observed in this group.

Representative gross morphology at necropsy is shown in Figure 9. HFD + vehicle mice displayed markedly increased visceral fat accumulation in the inguinal and epididymal depots compared with ND + vehicle mice, consistent with the significantly elevated relative organ weights observed in this group. Treatment with silymarin (200 mg/kg), CPT (500 mg/kg), and low-dose CPTX (250 mg/kg) did not produce visible changes in visceral adiposity relative to the HFD + vehicle group. High-dose CPTX (500 mg/kg)-treated mice showed a qualitative reduction in visceral fat deposition, consistent with the significant reduction in relative liver weight observed in this group (Figure 5).

## 3. Discussion

Solvent fractionation of Chatuphalatika aqueous extract (CPT) yielded an ethyl acetate fraction (CPTX) with the highest phenolic content and the strongest HMG-CoA reductase inhibitory activity. In HFD-fed mice, CPTX improved serum lipid profiles, fasting blood glucose, serum ALT, and relative liver weight without affecting body weight or adipose tissue weights. These results show that the metabolic activity of Chatuphalatika is concentrated in a phenolic-enriched fraction with preferential hepatic effects, and that fractionation provides a more chemically tractable preparation than the crude decoction.

The enrichment of chebulagic acid in CPTX relative to CPT and other fractions was the most notable compositional shift. Stronger HMG-CoA reductase inhibition in CPTX paralleled this enrichment, consistent with the view that the lipid-regulatory activity of Chatuphalatika is associated with its phenolic matrix. CPTX thus provides a more chemically defined starting point for mechanistic and formulation studies.

Chatuphalatika shares three of its four constituent fruits with Triphala. Triphala has been shown to attenuate visceral adiposity in HFD-fed mice [13], and a systematic review suggests it may improve lipid profiles and fasting glucose, though clinical evidence remains limited [10]. Chatuphalatika has reported anti-inflammatory and antihyperuricemic activity [11], consistent with properties attributed to Triphala [12], reflecting their shared botanical composition. The present study extends this background by identifying a phenolic-enriched fraction as the active component of Chatuphalatika, a level of chemical resolution not previously reported for this formulation.

CPTX inhibited HMG-CoA reductase at 83.9% (1 µg/mL), stronger than the 36.4% reported for Triphala aqueous extract at 5 µg/mL [14]. This is consistent with molecular docking data showing that tannin-type compounds including chebulagic acid and chebulinic acid interact with the HMG-CoA reductase active site [18], and with experimental evidence that Triphala aqueous extract inhibits HMG-CoA reductase and reduces TC, TG, and LDL-C in rats [14]. The reductions in serum TC, TG, and LDL-C in CPT- and CPTX-treated mice are in line with this mechanism. However, the in vitro assay does not capture the full complexity of hepatic cholesterol regulation in vivo; parameters such as HMG-CoA reductase expression, hepatic cholesterol content, LDL receptor activity, and fecal sterol output were not assessed, and contributions from altered lipid absorption, bile acid metabolism, or hepatic lipid handling cannot be excluded.

Chebulagic acid was the most enriched constituent in CPTX and serves as a useful chemical marker. However, purified chebulagic acid was not tested individually, so its isolated contribution cannot be determined. CPTX also contains gallic acid, ellagic acid, and chebulinic acid, each with reported hepatic and metabolic activities [19,20]. Gallic acid has hepatoprotective effects through antioxidant and anti-inflammatory mechanisms [19], and ellagic acid improves hepatic lipid metabolism in mice [20]. Synergistic antioxidant interactions among the four constituent fruits of Chatuphalatika have been reported [17], and chebulagic acid has been shown to suppress TNF-α-induced NF-κB and MAPK activation in retinal endothelial cells in vitro [21], suggesting a possible anti-inflammatory mechanism relevant to the hepatic effects observed here. The activity of CPTX is therefore best attributed to its enriched phenolic matrix rather than any single compound. Phenolic constituents were quantified by HPLC-PDA against authentic standards, which reliably quantifies the targeted compounds but does not provide the structural confirmation afforded by mass spectrometry; untargeted profiling by LC-HR-ESI-MS/MS would allow more comprehensive characterization of the hydrolyzable tannins and chebulagic acid in CPTX and is recommended for future work.

The in vivo experiment was conducted under continued HFD feeding, so the metabolic improvements reflect activity under sustained dietary challenge. Under these conditions, CPTX consistently improved TC, TG, LDL-C, and HDL-C at both doses. Comparable lipid-lowering effects have been reported for constituent fruits of related formulations. *Terminalia bellirica* extract improved hyperlipidemia and insulin resistance in spontaneously obese diabetic mice [22], and *Terminalia chebula* methanolic bark extract reduced serum lipids in HFD-induced hyperlipidemic rats [23], though differences in plant part and extraction method limit direct comparison. HDL-C interpretation requires caution: murine lipoprotein metabolism is HDL-dominated and differs from the human profile, so the lipid effects of CPTX are more meaningfully interpreted through reductions in TC, TG, and LDL-C, with HDL-C as a supporting endpoint.

CPT improved serum lipid profiles despite being the unfractionated decoction, consistent with previously reported activity [15]. CPTX retained this lipid-modulating activity and additionally reduced fasting blood glucose and relative liver weight at 500 mg/kg. The two preparations showed comparable effects on lipid parameters; the additional benefits of CPTX were specific to glucose and liver weight at the higher dose. This suggests that the hepatic and glucose-related effects require a higher or more selective phenolic exposure than what is present in the crude extract. CPT and CPTX thus represent different levels of phytochemical refinement: CPT reflects the broader activity of the traditional preparation, while CPTX provides a more defined hepatic profile.

High-dose CPTX reduced relative liver weight while body weight, iWAT, and eWAT remained unchanged. In HFD models, increased liver weight reflects hepatic lipid accumulation and metabolic dysfunction [24]; its reduction in CPTX-treated animals suggests improved hepatic metabolic status rather than systemic fat loss. Serum ALT was reduced in both CPT- and CPTX-treated groups, consistent with a hepatoprotective effect. These results align with reports that *Phyllanthus emblica* extract attenuates HFD-induced hepatic steatosis in rodent NAFLD models [25,26] and that chebulagic acid normalizes ALT, antioxidant enzymes, and inflammatory cytokines in CCl_4_-induced hepatic fibrosis [27]. However, hepatic histology, hepatic triglyceride content, and molecular markers were not assessed; the reductions in liver weight and ALT should therefore be interpreted as indicators of improved hepatic metabolic status, not as confirmation of reduced steatosis.

The coordinated improvements in serum lipids, fasting glucose, ALT, and liver weight observed in CPTX-treated animals are consistent with the broad metabolic activity attributed to hydrolyzable tannins and their gut-derived metabolites. Ellagitannins undergo hydrolysis and microbial biotransformation to generate ellagic acid and urolithin derivatives, compounds with documented antioxidant, anti-inflammatory, lipid-regulatory, and glucose-modulating properties [20,28,29]. At the same time, the present study did not assess pharmacokinetic parameters, the extent of gut microbial transformation, or downstream hepatic signaling. The relative contributions of intact tannins and their metabolites to the observed effects therefore remain to be determined. A further limitation is the absence of biochemical measurements at the end of the induction period. Direct confirmation of metabolic syndrome prior to treatment was therefore not obtained; however, the persistent elevation of serum lipids, fasting glucose, and ALT in the untreated HFD group at study termination indicates that the metabolic phenotype was maintained throughout.

High-dose CPTX reduced fasting blood glucose in HFD-fed mice. Polyphenols have been reported to improve glucose homeostasis through oxidative stress reduction, hepatic glucose metabolism, and insulin signaling modulation [10,28]. Chebulagic acid improved glucose metabolism via PPARγ and GLUT4 upregulation in a streptozotocin-induced diabetic model [30], though this model differs mechanistically from HFD-induced insulin resistance, and direct extrapolation is limited. Since only fasting blood glucose was measured (insulin levels, glucose tolerance, and insulin resistance indices were not evaluated), the finding should be interpreted as reduced fasting glucose disturbance rather than improved insulin sensitivity. The dose-dependence pattern, with lipid and ALT effects at both doses but glucose and liver weight effects only at 500 mg/kg, suggests these endpoints may have different phenolic exposure thresholds.

Silymarin was included as a reference hepatoprotective agent. It has reported anti-obesity and hepatoprotective activity through farnesyl X receptor activation [24,31], and in this study it improved serum lipid parameters in HFD-fed mice. High-dose CPTX additionally reduced fasting blood glucose and relative liver weight, endpoints not significantly changed by silymarin. Unlike silymarin, high-dose CPTX showed significant reduction in fasting blood glucose and relative liver weight under the present conditions. Whether this reflects a genuine pharmacological difference requires a head-to-head comparison.

CPTX concentrates the metabolic activity of Chatuphalatika into a more chemically defined fraction. The combination of phytochemical characterization, HMG-CoA reductase inhibition, and in vivo results supports phenolic enrichment as a meaningful step toward identifying the active component of this formulation. Improvements in dyslipidemia, fasting glucose, ALT, and liver weight without changes in body weight or adipose mass point to a predominantly hepatic mechanism. CPTX may serve as a starting point for further development, but safety evaluation, pharmacokinetic data, and mechanistic studies are required before any clinical relevance can be established.

## 4. Materials and Methods

### 4.1. Plant Materials

Dried fruits of *Terminalia chebula*, *Terminalia bellirica*, *Terminalia arjuna*, and *Phyllanthus emblica* were purchased from Tong In Drugstore, Maha Sarakham, Thailand. The plant materials were authenticated based on macroscopic characteristics according to the Thai Herbal Pharmacopoeia. Voucher specimens of *T. chebula* (MSU.PH-COM-TC05), *T. bellirica* (MSU.PH-COM-TB05), *T. arjuna* (MSU.PH-COM-TA03), and *P. emblica* (MSU.PH-EUP-PE05) were deposited at the Faculty of Pharmacy, Mahasarakham University, Maha Sarakham, Thailand.

### 4.2. Preparation of Chatuphalatika Aqueous Extract and Solvent Fractions

The dried fruits were pulverized into powder and passed through a No. 18 mesh sieve. Equal amounts of each powdered plant material (150 g each) were thoroughly mixed to obtain the Chatuphalatika herbal mixture. The mixture was boiled with 10 L of distilled water for 1 h to prepare the decoction, corresponding to the traditional method of preparation. The decoction was filtered, and the filtrate was divided into two portions.

One portion was freeze-dried using a freeze dryer (ScanVac, Allerød, Denmark) to obtain the Chatuphalatika aqueous extract, hereafter referred to as CPT. The remaining portion was subjected to sequential liquid–liquid partitioning with solvents of increasing polarity, including n-hexane, chloroform, and ethyl acetate. The obtained solvent fractions were concentrated under reduced pressure using a rotary evaporator (Heidolph, Schwabach, Germany). The ethyl acetate fraction, which contained the highest phenolic content, was designated as CPTX. All dried extracts and fractions were stored at −20 °C until further analysis. The percentage yield of each extract or fraction was calculated relative to the initial dry extract weight.

### 4.3. Determination of Total Phenolic Content

The total phenolic content of CPT and its solvent fractions was determined using the microplate Folin–Ciocalteu colorimetric method [32]. Briefly, appropriately diluted sample solutions were reacted with Folin–Ciocalteu reagent and alkaline solution according to the assay protocol. Absorbance was measured at 630 nm using a microplate reader (BMG Labtech, Ortenberg, Germany). Gallic acid was used as the reference standard, and the calibration curve was prepared using known concentrations of gallic acid. The linear regression equation was y = 0.003x + 0.0546, with a correlation coefficient of R^2^ = 0.999. The total phenolic content was expressed as milligrams of gallic acid equivalents per gram of extract or fraction (mg GAE/g extract). All measurements were performed in triplicate.

### 4.4. HPLC Analysis of Phenolic Constituents

The phenolic constituents of CPT and its solvent fractions were analyzed by high-performance liquid chromatography (HPLC) as previously described [11]. Gallic acid, ellagic acid, chebulagic acid, and chebulinic acid were used as reference standards. All standard compounds were purchased from Sigma-Aldrich (St. Louis, MO, USA). Stock solutions of each standard were prepared in methanol at a concentration of 1 mg/mL and subsequently diluted with methanol to obtain the desired concentrations for calibration. The extracts and fractions were dissolved in methanol at a concentration of 1 mg/mL before analysis.

Chromatographic separation was performed using a Shimadzu SCL-10A VP HPLC system equipped with LC-10AD binary pumps and an SPD-M20A photodiode array detector (Shimadzu, Kyoto, Japan). Data acquisition and processing were conducted using Class-VP software version 6.1. Separation was achieved on an Eclipse XDB-C18 column (250 × 4.6 mm, 5 µm) at ambient temperature. The mobile phase consisted of 0.05% trifluoroacetic acid in water (solvent A) and acetonitrile (solvent B). The mobile phases were filtered through a 0.45 µm membrane filter and degassed before use. The flow rate was maintained at 1.0 mL/min. Gradient elution was performed as follows: 0–2 min, 5% B; 2–4 min, 5–10% B; 4–12 min, 10–15% B; 12–26 min, 15–30% B; 26–30 min, 30–100% B; 30–35 min, 100% B; 35–40 min, 100–5% B; and 40–45 min, 5% B. The injection volume was 20 µL, and the detection wavelength was set at 270 nm. Phenolic compounds were identified by comparing their retention times and UV spectra with those of authentic standards. Quantification was carried out using external calibration curves constructed from corresponding reference standards. All samples were analyzed in triplicate, and the contents of phenolic constituents were expressed as milligrams per gram of extract or fraction (mg/g extract).

### 4.5. HMG-CoA Reductase Inhibition Assay

The inhibitory activity of CPT and its solvent fractions against 3-hydroxy-3-methylglutaryl coenzyme A reductase (HMG-CoA reductase) was evaluated using a commercial HMG-CoA reductase assay kit (Sigma-Aldrich, USA) according to the manufacturer’s instructions. Sample solutions were freshly prepared before analysis. Pravastatin was used as the positive control. The enzymatic reaction was monitored spectrophotometrically by measuring the decrease in absorbance at 340 nm, corresponding to NADPH oxidation during the HMG-CoA reductase-catalyzed reaction. The percentage inhibition of HMG-CoA reductase activity was calculated relative to the untreated enzyme control. All measurements were performed in triplicate.

### 4.6. Animal and Experimental Design

Male C57BL/6NJcl mice, aged 6–7 weeks and weighing 20–22 g, were obtained from Nomura Siam International Co., Ltd. (Bangkok, Thailand). A total of 60 mice were used in this study. After one week of acclimatization, the mice were assigned to six experimental groups, with 10 mice per group (n = 10/group) as follows: normal diet plus vehicle (ND + vehicle), high-fat diet plus vehicle (HFD + vehicle), HFD plus silymarin 200 mg/kg (HFD + SLM 200), HFD plus CPT 500 mg/kg (HFD + CPT 500), HFD plus CPTX 250 mg/kg (HFD + CPTX 250), and HFD plus CPTX 500 mg/kg (HFD + CPTX 500).

Mice in the HFD groups were fed a high-fat diet for 20 weeks, and treatments were administered orally once daily during the final 8 weeks while HFD feeding was continued. All animal procedures were approved by the Institutional Animal Care and Use Committee of Mahasarakham University (IACUC-MSU-019/2020). The animals were housed under controlled environmental conditions at 22 ± 2 °C, 50 ± 10% relative humidity, and a 12 h light/dark cycle, with free access to food and water.

Silymarin (product specifications: 80% silymarin by UV-VIS, 30% silybin by HPLC, and 40% silymarin water-soluble form; Naturalin Bio-Resources Co., Ltd., Changsha, China; NAT-145; *Silybum marianum* (L.) Gaertn., Asteraceae) was used as a reference hepatoprotective agent at a dose of 200 mg/kg. The doses of CPT and CPTX were selected based on preliminary experiments and previous studies describing the metabolic effects of polyphenol-rich plant extracts. Body weight and food intake were recorded throughout the experimental period. Caloric intake was calculated based on the amount of food consumed and the energy density of each diet. At the end of the experiment, mice were fasted overnight and anesthetized. Blood samples were collected for biochemical analysis. The liver, inguinal white adipose tissue (iWAT), and epididymal white adipose tissue (eWAT) were excised, weighed, and expressed as relative organ weights using the following equation:

Relative organ weight (%) = [organ weight (g)/body weight (g)] × 100

### 4.7. Biochemical Analysis

After overnight fasting, fasting blood glucose levels were measured using a glucometer and test strips (Accu-Chek, Roche Diabetes Care GmbH, Mannheim, Germany). Blood samples were then collected and centrifuged to obtain serum. Serum levels of total cholesterol (TC), triglycerides (TG), high-density lipoprotein cholesterol (HDL-C), aspartate aminotransferase (AST), and alanine aminotransferase (ALT) were measured using commercial diagnostic kits (DiaSys Diagnostic Systems, GmbH, Holzheim, Germany) and an automated chemistry analyzer (Sysmex BX-3010, Sysmex Asia Pacific Pte Ltd., Singapore). Low-density lipoprotein cholesterol (LDL-C) was calculated using the Friedewald equation [33]:

LDL-C = TC − HDL-C − TG/5

where TC, HDL-C, and TG values are expressed in mg/dL.

### 4.8. Statistical Analysis

Data are presented as mean ± SEM for animal experiments and as mean ± SD for phytochemical and enzyme inhibition assays, unless otherwise indicated. Statistical analysis was performed using SigmaPlot software version 11.0 (Systat Software Inc., San Jose, CA, USA). For two-group comparisons (ND vs. HFD during the induction period), Student’s *t*-test was used. For multi-group comparisons during the treatment period, one-way analysis of variance (ANOVA) followed by the Student-Newman-Keuls post hoc test was applied. For the HMG-CoA reductase inhibition assay, one-way ANOVA followed by Tukey’s post hoc test was used. A value of *p* < 0.05 was considered statistically significant.

## 5. Conclusions

Solvent fractionation of Chatuphalatika aqueous extract produced an ethyl acetate fraction (CPTX) enriched in hydrolyzable tannins, particularly chebulagic acid. CPTX showed the strongest HMG-CoA reductase inhibitory activity and produced the most consistent improvements in HFD-induced metabolic disturbances. It improved serum lipid profiles, fasting blood glucose, relative liver weight, and ALT more than the parent extract and other fractions, indicating that phenolic enrichment enhances the metabolic efficacy of Chatuphalatika.

The reduction in relative liver weight without changes in body weight or adipose tissue points to a predominantly hepatic effect, possibly involving modulation of cholesterol biosynthesis, hepatic lipid accumulation, and inflammatory or oxidative stress pathways. The activity of CPTX is likely attributable to its enriched phenolic matrix rather than any single compound.

These findings provide experimental support for Chatuphalatika as a phenolic-rich formulation with metabolic relevance to dyslipidemia and hepatic dysfunction. As a further development direction, the phenolic-enriched fraction identified here could inform optimization of extraction and formulation processes, such as the microwave-assisted extraction and direct-compression tablet approach recently reported for Chatuphalathika [34], to produce a standardized phenolic-rich preparation. CPTX warrants further investigation, but mechanistic studies, pharmacokinetic evaluation, and clinical data are required before conclusions about therapeutic applicability can be drawn.

## Figures and Tables

**Table 1:** Extraction yield, total phenolic content, and major phenolic constituents of Chatuphalatika aqueous extract (CPT) and its solvent fractions.

Samples	Yield (%)	Total Phenolic Content (mg GAE/g Extract)	Gallic Acid (mg/g Extract)	Ellagic Acid (mg/g Extract)	Chebulagic Acid (mg/g Extract)	Chebulinic Acid (mg/g Extract)
CPT	22.86	363.0 ± 13.33	99.8 ± 5.62	7.2 ± 0.44	50.9 ± 0.94	27.2 ± 1.32
Hexane	Negligible ^a^	ND	ND	ND	ND	ND
Chloroform	11.76	6.6 ± 0.42	4.4 ± 0.43	0.5 ± 0.02	1.9 ± 0.07	1.4 ± 0.17
Ethyl acetate (CPTX)	27.21	458.8 ± 25.96	72.0 ± 4.33	5.3 ± 0.82	105.5 ± 4.72	25.4 ± 1.46
Aqueous	37.10	113.0 ± 5.50	6.5 ± 0.13	2.9 ± 0.43	13.7 ± 0.18	12.5 ± 0.39

Values are expressed as mean ± SD (n = 3). GAE, gallic acid equivalent; ND, not detected. ^a^ Yield of the hexane fraction was negligible and was not weighed due to insufficient quantify.

## Data Availability

The original contributions presented in this study are included in the article. Further inquiries can be directed to the corresponding author.
